# Effects of midwife-led maternity services on postpartum wellbeing and clinical outcomes in primiparous women under China’s one-child policy

**DOI:** 10.1186/s12884-018-1969-9

**Published:** 2018-08-13

**Authors:** Jing Hua, Liping Zhu, Li Du, Yu Li, Zhuochun Wu, Da Wo, Wenchong Du

**Affiliations:** 1 Shanghai First Maternity and Infant Hospital, Tongji University School of Medicine, P.O. 2699 Gaoke Road, Shanghai, 200042 China; 2 Shanghai Maternity and Child Health Care Center, P.O. 339 Gaoke Road, Shanghai, 200042 China; 3 Health Statistics and Social Medicine Department of Public health School, Fudan University, Shanghai, 200002 China; 4 Research Center for Translational Medicine, East Hospital, Tongji University School of Medicine, Shanghai, 200002 China; 5 Division of Psychology, Nottingham Trent University, Chaucer Building 4013, Burton Street, Nottingham, NG1 4BU UK

**Keywords:** Midwife-led maternity services, Postpartum wellbeing, China’s one-child policy

## Abstract

**Background:**

The Midwife-led maternity services have been implemented in China in response to the high rates of primiparous women and Caesarean Sections (CS) which may be related to China’s one-child policy. However, few studies in China have been reported on the effectiveness of Midwife-led Care at Delivery (MCD) and the Continuity of Midwife-led Care (CMC) on postpartum wellbeing and other clinical outcomes. Therefore, evidence-based clinical validation is needed to develop an optimal maternity service for childbearing women in China.

**Methods:**

A concurrent cohort study design was conducted with 1730 pregnant women recruited from 9 hospitals in Shanghai. Among the 1730 participants at baseline, 1568 participants completed the follow-up questionnaire, with a follow-up rate of 90.6%.

**Results:**

Compared with the routine Obstetrician-led Maternity Care (OMC), Midwife-led Care at Delivery (MCD) was associated with CS rate (OR were 0.16; 95%CI: 0.11 to 0.25) and a higher total score of postpartum wellbeing (βwere 2.70; 95%CI: 0.70 to 4.70) when adjusting for the baseline differences and other confounders during delivery or postpartum period. Moreover, continuity of Midwife-led Care (CMC) was associated with CS rate (OR were 0.30; 95%CI: 0.23 to 0.41), as well as increased rate of breastfeeding within the first 24 h (OR were 2.49; 95% CI: 1.47 to 4.23), higher postpartum satisfaction (β = 4.52; 95% CI: 1.60 to 12.68), lower anxiety (βwere 0.66; 95% CI: 0.16 to 1.17), increased self-control (βwere 0.39; 95% CI: 0.02 to 0.76) and a higher total score of postpartum wellbeing (βwere 3.14; 95% CI: 1.54 to 4.75).

**Conclusion:**

CMC is the optimal service for low-risk primiparous women under China’s one-child policy, and is worthwhile for a general implementation across China.

## Background

There are many different models of maternity care available for looking after the health and wellbeing of pregnant women and newborns during pregnancy, labour and postpartum, each with their own distinct features based on local, cultural or social traditions and knowledge. Usually the obstetrician is the lead healthcare professional, but other times a midwife might be in charge. This responsibility can also be shared between obstetricians and midwives, while in other instances the midwife is only involved during labour (Midwife-led Care at Delivery, MCD). One of the recent models is called the Continuity of Midwife-led Care (CMC), where the midwife is responsible for the health care of pregnant women from the initial booking appointment, through labour and all the way to postpartum.

Research have indicated that midwife-led models of care are associated with improved benefits for mothers and newborns, including shorter labour, and decreased likelihood to require intrapartum analgesia or report dissatisfaction with their childbirth experiences [1–3], which are typical indicators of optimal maternity care for low-risk childbearing women [4]. Midwife-led care facilitates birth, provides a better memory of the experience for the mothers, reduces or eliminates the need for medical intervention [5], decreases the number of maternal requested Caesarean Sections (CS) [6–8] and helps pregnant women form a strong bond with their midwives. Midwife-led care may also lead to improved maternal psychosocial outcomes [9–11], whereby pregnant women reported a stronger sense of emotional support, reassurance and were more in control during the midwives’ antenatal care [12]. Midwives who offer continuity of care can also provide better support, information and guidance for the feeding of newborns [3, 4, 13, 14].

China’s one-child family policy was introduced in 1979, restricting couples in urban areas to have only one child. Under the policy, most Chinese women are primiparous, and hence lack the experience of pregnancy and postpartum period. Moreover, the parents or parents-in-law of childbearing women are culturally expected to take the responsibility of caring for the new mothers and newborns from pregnancy to postpartum period. However, they may also lack the relevant experience because they have had only one child themselves under the population control policy and therefore may only be able to provide very limited assistance. Notably, due to the implementation of China’s one-child policy [15], there has been a dramatic increase in the overall rate of CS (54.90% throughout mainland China) over the past ten years [16, 17], and the most common reported indication for CS (28.43%) was maternal request for none-medical reasons [18]. Therefore, a midwife who can provide consultation, birth planning (encouraging low-risk pregnant women to choice vaginal birth if their health conditions are permissible), parent education and psychological support to inexperienced primiparous pregnant women from childbirth through to postpartum period, can play an important role for primiparous women under the one-child policy [19].

However, current maternity services in China are predominantly hospital-based, with the obstetrician as the lead professional in routine antenatal checkup [20]. Recently, some Chinese hospitals have started to introduce the midwife-led care model [21–23], which generally provides one-to-one midwife-led care at delivery. A small proportion hospitals provides intentional continuity of midwife-led care from pregnancy to postpartum period [17, 24] Researchers have conducted pilot studies and reported that compared to the obstetrician-led antenatal care, midwife-led care is an effective way to reduce CS rate for childbearing women in mainland China [20]. Recently, an intervention study conducted in China showed that the midwife-led continuity care model decreased the CS rate and improved women’s general satisfaction [17] compared with obstetrician-led antenatal care. To our knowledge, few studies with clinical validation have reported on the effectiveness of Midwife-led Care at Delivery (MCD) and the Continuity of Midwife-led Care (CMC), which is needed in order to develop an optimal maternity service for childbearing women under China’s one-child policy. We therefore conducted a concurrent cohort study on maternal psychological outcomes in Shanghai, China. We hypothesized that midwife-led maternity care is an effective way to improving maternal satisfaction and wellbeing (i.e. general health, self-control, and vitality), reducing CS rates, and increasing the rate of breastfeeding. The continuity of midwife maternity care may be an optimal service for women due to their inexperience of maternity care under China’s one-child policy. The aim of this study was: (1) to explore the effects of MCD and CMC on the delivery mode and rate of breastfeeding within the first 24 h in primiparous women when compared with Obstetrician-led Maternity Care (OMC) under China’s one-child policy; (2) to compare the effectiveness of MCD and CMC with routine maternity care on postpartum satisfaction and wellbeing so as to provide the evidence for selecting an optimal maternity service in China.

## Methods

### Participants

**Fig. 1:**
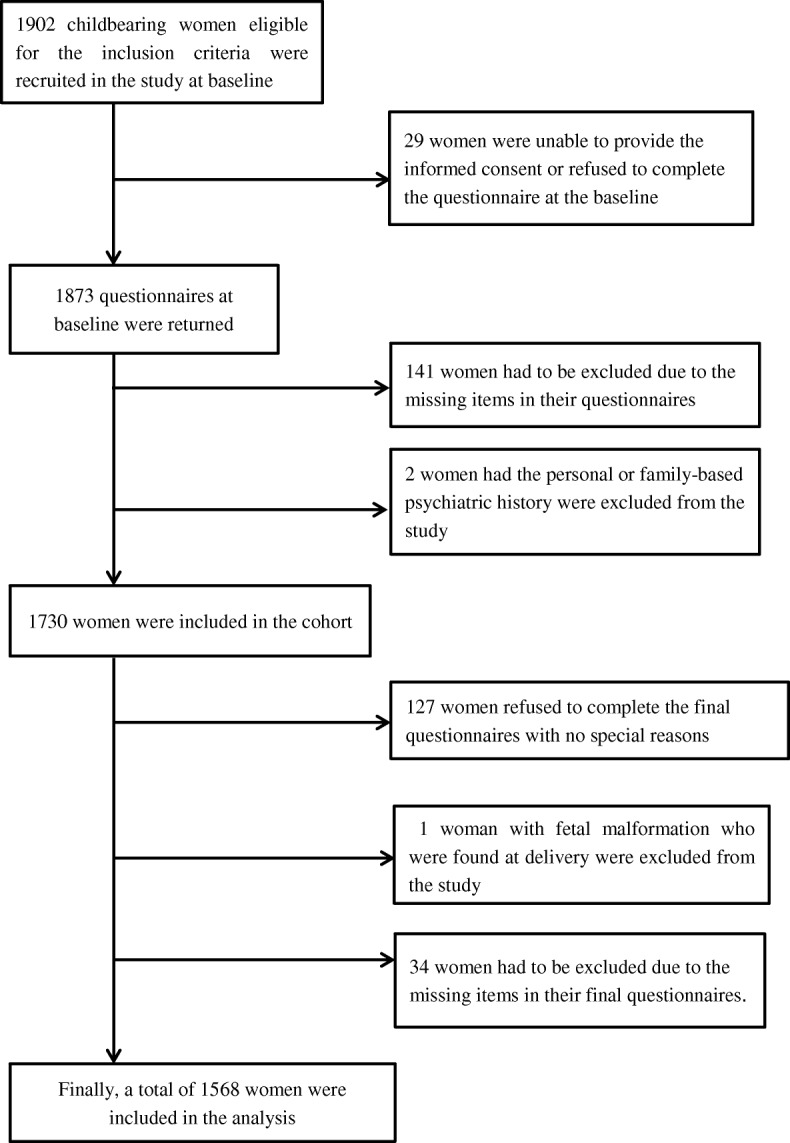
Number of women who completed the baseline and follow-up investigations

A concurrent cohort study design (a follow-up study that compares outcomes between participants who have received an intervention and those who have not) was used to collect data at two time points (baseline and follow-up). In order to provide a better representation of sample data in this study, we randomly selected 8 hospitals from 8 separate districts across Shanghai. Throughout May 2013, Chinese childbearing women who attended the antenatal clinics of the 8 selected hospitals were eligible for the trial if they met the following inclusion criteria: (1) singleton pregnancy; (2) primiparous; (3) 29–30 weeks of gestation at recruitment; (4) absence of medical or obstetric complications. The exclusion criteria were: (1) fetal malformations; (2) severe personal, family-based psychiatric or medical history; (3) unable to provide consent. A total of 1902 childbearing women who were eligible based on the inclusion criteria were invited to take part in the study at baseline; 29 women were unable to provide the informed consent or refused to complete the questionnaire. A total of 1730 childbearing women were included in the cohort study, and finally 1568 women who completed the follow-up questionnaire with no missing information and had no fetal malformation were included in the final analysis (Fig. 1).

### Measurements

The General Wellbeing Schedule (GWBS) was used to assess maternal wellbeing at baseline and follow-up. GWBS is an 18-item multi-dimensional scale developed by Dr. Harold Deputy and revised by Frazio [25]. It covers six dimensions of subjective feelings of psychological wellbeing: without anxiety (the 10th and 15th items), without depression (the 4th, 12th, and 18th items), general health (the 2nd, 5th, 8th, 16th items), positive wellbeing (the 6th and 11th items), self-control (the 3rd, 7th, 13th items), and vitality (1st, 9th, 14th, and 17th items). The GWBS scale has been translated into Chinese and have since been validated in China [26]; the internal consistency (Cronbach’s alpha coefficients of male and female were 0.91 and 0.95 respectively) and construct validity (Pearson correlation coefficients between the scores of each item and total score were from 0.65 to 0.88) of GWBS in Chinese have been reported to be reliable. A higher score reflected the increased maternal wellbeing.

A self-reported questionnaire was developed to measure women’s demographic and clinical characteristics at baseline [27]. The satisfaction with childbirth experiences in prenatal and perinatal care was assessed using a self-reported follow-up questionnaire. The follow-up questionnaire also inquired about the women’s mode of delivery, time of gestation at delivery, whether breastfeeding was commenced within 24 h, and presence of newborn malformations [27].

### Model of maternity service

In OMC model, obstetricians are the primary providers of maternal care to pregnant women. However, because pregnant women are required to visit the obstetrician’s antenatal clinic at the out-patient department, it is likely that they would have met a different obstetrician on each visit. Furthermore, when the pregnant women came into the hospital for labour, they would also be cared for by an obstetrician who was rostered for duty on that day.

In the CMC model, the midwives (usually undertaken by nurses who hold certification from the local health bureau and have received specific post-nursing training in midwifery for 1 to 3 years, as well as passed the qualification test) provide assistance to healthy women with low-risk pregnancies. Thus, a small group of midwives are usually responsible for providing care to the pregnant women including antenatal checkups, consultation, birth planning, parenting education. They are also responsible for providing care during labour, birth and the immediate postpartum period, and collaborating with obstetricians and other health professionals. The advantages of this midwife-led plan include providing continuous emotional support to the pregnant women, thereby minimizing intra-partum fetal monitoring, offering alternatives to pain relief (pharmacological or non-pharmacological), providing different positions that allow free movement, reduction of episiotomy, and promoting mother and child bonding such as facilitating skin-to-skin contact between mother and baby, as well as encouraging breastfeeding after birth. However, childbearing women also have the right to select the other model (MCD) at their own discretion. In the MCD model, the pregnant woman would visit the obstetrician for consultation during pregnancy, and thereafter receive one-to-one care by a designated midwife from the onset of labour to 2 h postpartum.

### Procedure

The survey was conducted from May to October 2010. The questionnaire at baseline was filled out by the pregnant women when they attended regular health education courses conducted by hospitals. We conducted the follow-up investigation on day 7 postpartum. Women were asked to fill out the questionnaire according to the instructions given by the researchers of the study.

### Statistical analysis

Data were analyzed using the SPSS 17.0 program. Independent-samples t tests and Chi-square tests performed to compare the 2 groups at the baseline. Multiple linear regression analyses were used to assess the effects of MCD and CMC on delivery mode and maternal satisfaction, after adjusting for hospitals, baseline differences and the other confounders during birth. All variables were simultaneously included in the model. Because the Variance Inflation Factor (VIF) of each predictor in the model was less than 10, the multicolinearity was not statistically significant. Therefore, the colinearity was not considered in the model. Multiple logistic regression models were used to analyze the effects of MCD and CMC on maternal wellbeing after adjusting for hospitals, baseline differences and other confounders during birth. A value of 0.05 was used for all tests of significance.

## Results

### Baseline

Of the 1730 participants at baseline, 1568 participants completed the follow-up questionnaire (Fig. 1), with a follow-up rate of 90.6%. All women were allowed to choose their preferred type of care (MCD, CMC or OMC). Among these participants, 279 (17.8%) women selected the MCD, 172 (10.1%) women selected the CMC, and 1117 (71.1%) selected the routine obstetrician-led care birth. Table 1 shows the distribution of selected characteristics among participants who chose the different birth-care services. At cohort entry, the proportions of maternal age, and multiple gestation was equal (each *P* > 0.05; Table 1), whereas the proportions of ethnicity, education, occupation, and method of payment were not equal (each *P* < 0.05; Table 1). However, the degree of maternal wellbeing (non-anxiety, non-depression, positive wellbeing and self-control) in the two birth-care services were significantly different with the routine OMC group (each *P* < 0.05; Table 2).

**Table 1:** Demographic and clinical characteristic of participants at baseline

Characteristic	Total *n* = 1568	OMC^a^ group *n* = 1117	MCD^b^ group *n* = 279	CMC^c^ group *n* = 172	*P*
Maternal age [M(SD)] ^d^	28.52 (3.59)	28.47 (3.74)	28.12 (3.33)	29.53 (2.77)	< 0.001***
Ethnicity n(%)^e^					
Han	1503 (95.9)	1069 (95.7)	266 (95.3)	168 (97.7)	0.431
Non-Han	65 (4.1)	48 (4.3)	13 (4.7)	4 (2.3)	
Education *n*(%)^e^					
College or university	1173 (74.8)	832 (74.5)	176 (63.1)	16,595.9	< 0.001***
Middle school	303 (19.4)	224 (20.1)	73 (26.2)	6 (3.9)	
Primary school or below	92 (5.8)	61 (5.5)	30 (10.8)	1 (0.2)	
Vacation *n*(%)^e^					
Company employee	338 (21.6)	233 (20.9)	82 (29.4)	23 (13.4)	< 0.001***
Private owner	108 (6.9)	87 (7.8)	12 (4.3)	9 (5.2)	
Technician and liberal profession	642 (40.9)	449 (40.2)	96 (34.4)	97 (56.4)	
Unemployed	291 (18.6)	210 (18.8)	61 (21.9)	20 (11.6)	
Others	189 (12.1)	138 (12.4)	28 (10.0)	23 (13.4)	
Multiple Gestation *n*(%)^e^					
No	1171 (74.7)	855 (76.5)	194 (69.5)	122 (72.6)	0.055
Yes	397 (25.3)	266 (23.5)	85 (30.5)	46 (27.4)	
Method of payment *n*(%)^e^					
Self-payment	512 (32.7)	368 (32.9)	120 (43.0)	24 (14.0)	0.001**
Government insurance	707 (45.1)	501 (44.9)	105 (37.6)	101 (58.7)	
Private insurance	349 (22.3)	248 (22.2)	54 (19.4)	47 (27.3)	

^a^Obstetrician-led Maternity Care

^b^Midwife-led Care at Delivery

^c^Continuity of Midwife-led Care

^d^One-way ANOVA

^e^Pearson’s chi-squared test

*p < 0.05, **p < 0.01,***p < 0.001

**Table 2:** Maternal wellbeing of participants at baseline

Characteristic	Total *n* = 1568	OMC^a^ group *n* = 1117	MCD^b^ group *n* = 279	CMC^c^ group *n* = 172	*P*
Maternal wellbeing [M(SD)] ^d^					
Anxiety	7.72 (2.32)	7.63 (2.50)	8.01 (2.53)	7.85 (2.30)	< 0.001***
Depression	16.12 (6.35)	16.39 (6.05)	13.65 (8.16)	18.41 (6.44)	< 0.001***
General health	19.04 (4.67)	19.11 (4.63)	17.72 (5.10)	20.77 (4.51)	< 0.001***
Positive wellbeing	17.12 (2.37)	17.05 (2.31)	17.65 (2.86)	16.77 (2.63)	< 0.001***
Self-control	27.28 (2.72)	27.16 (2.66)	27.98 (3.29)	26.94 (2.67)	< 0.001***
Vitality	28.07 (4.02)	28.13 (3.96)	26.86 (4.33)	29.63 (3.98)	< 0.001***
Total score	115.36 (11.86)	115.48 (11.85)	112.12 (12.37)	119.85(10.33)	< 0.001***

^a^Obstetrician-led Maternity Care

^b^Midwife-led Care at Delivery

^c^Continuity of Midwife-led Care

^d^One-way ANOVA

*p < 0.05, **p < 0.01,***p < 0.001

### Delivery mode

Table 2 shows the associations of MCD and CMC with the delivery mode and rate of breastfeeding in the first 24 h. We found that odds of caesarean section when compared with OMC were decreased significantly with CMC (OR 0.30; 95% CI: 0.22 to 0.40) and with MCD (OR 0.17; 95% CI: 0.11 to 0.25). The rates of caesarean section in CMC (OR 0.30; 95%CI: 0.23 to 0.41) and MCD (OR 0.16; 95%CI: 0.11 to 0.25) group also decreased when adjusting for baseline differences and other confounders at delivery or postpartum period (Table 3).

**Table 3:** Effects of MCD and CMC on delivery mode and rate of breastfeeding in 24 h

Outcome variables	OMC^a^ group (%) *n* = 1117	MCD^b^ group (%) *n* = 279	CMC^c^ group (%) *n* = 172	MCD vs. OMC		CMC vs. OMC	
				cOR^d^ (95% CI)	aOR(95% CI)	cOR^d^ (95% CI)	aOR^e^ (95% CI)
Delivery mode n(%)							
Vaginal birth	461 (41.3)	139 (80.8)	196 (70.3)	Ref	Ref	Ref	Ref^e^
Caesarean section	656 (58.7)	33 (19.2)	83 (29.7)	0.17 (0.11,0.25)***	0.16 (0.11,0.25) ^e^***	0.30 (0.22,0.40)***	0.30 (0.23,0.41) ^e^***
Breastfeeding in first 24 h n(%)							
No	1006 (90.1)	233 (83.5)	136 (78.8)	Ref	Ref	Ref	Ref^f^
Yes	111 (9.90)	46 (16.5)	36 (21.2)	1.36 (0.96,1.93)	1.38 (0.97,1.96) ^f^	2.46 (1.46,4.13)**	2.49 (1.47,4.23) ^f^ **

^a^Obstetrician-led Maternity Care

^b^Midwife-led Care at Delivery

^c^Continuity of Midwife-led Care

^d^cOR indicates crude odds ratio

^e^aOR indicates adjusted odds ratio (adjusted for hospitals, maternal age, education, vacation, method of payment, gestational age at delivery and birth weight)

^f^aOR indicates adjusted odds ratio (adjusted for hospitals, maternal age, education, vacation, method of payment, mode of delivery, gestational age at delivery and birth weight)

*p < 0.05, **p < 0.01, ***p < 0.001

### Breastfeeding

The rate of breastfeeding in the first 24 h was only increased in the CMC group when the baseline differences and other confounders at delivery or postpartum period were adjusted for (OR 2.49; 95% CI:1.47, 4.23) or not adjusting for (OR 2.46; 95% CI: 1.46,4.13) (Table 3).

### Satisfaction

In our study, the satisfaction level increased only in the CMC group when the baseline differences and other confounders at delivery or postpartum period were adjusted for (OR 4.58; 95% CI: 1.63 to 12.86) or not adjusted for (OR 4.52; 95% CI: 1.60 to 12.68).

### Maternal wellbeing

We conducted the follow-up investigation at day 7 postpartum. The total score of postpartum wellbeing increased with MCD (β2.41; 95% CI:0.83 to 3.96) and CMC (β3.11; 95% CI:1.18 to 5.04) when confounders were not considered. The total score of wellbeing also increased with MCD (β2.70; 95% CI:0.70 to 4.70) and CMC (β3.14; 95% CI:1.54 to 4.75) after adjusting for the baseline differences and other confounders at delivery or postpartum period. The score of “anxiety” (a higher score indicating lower anxiety) was higher in CMC group when the baseline differences and other confounders were adjusted for (β0.66; 95% CI:0.16 to 1.17) or not adjusted for (β0.81; 95% CI:0.33 to 1.29). The score of “self-control” was also increased in CMC group when the baseline differences and other confounders were adjusted for (β0.39; 95% CI:0.02 to 0.76) or not adjusted for (β0.38; 95% CI:0.03 to 0.72) (Table 4).

**Table 4:** Effects of MCD and CMC on women’s postpartum wellbeing

Outcome variables	OMC^a^ group *n* = 1117 M(SD)	MCD^b^ group *n* = 451 M(SD)	CMC^c^ group *n* = 172 M(SD)	MCD vs. OMC		CMC vs. OMC	
				β^d^ (95% CI)	β^e^ (95% CI)	β^d^ (95% CI)	β^e^ (95% CI)
Anxiety(the higher score the less anxiety)	7.77 (4.26)	8.14 (3.03)	8.28 (2.75)	0.14 (−0.25,0.54)	0.07(−0.34,0.47)	0.81 (0.33,1.29)**	0.66 (0.16,1.17) *
Depression(the higher score the less depression)	18.79 (2.88)	18.83 (2.74)	18.95 (2.85)	0.04(−0.34,0.41)	0.20(−0.18,0.59)	0.16(− 0.29,0.62)	0.19(− 0.29,0.67)
General health	21.55 (3.70)	21.18 (4.05)	21.39 (3.87)	−0.38(− 0.88,0.12)	−0.12(− 0.62,0.39)	−0.17(− 0.77,0.44)	−0.16(− 0.80,0.50)
Positive wellbeing	16.97 (1.74)	16.82 (1.88)	17.23 (1.73)	−0.15(− 0.38,0.08)	−0.12(− 0.36,0.12)	0.25(− 0.03,0.54)	0.27(− 0.03,0.57)
Self-control	26.95 (2.11)	26.80 (2.51)	27.33 (1.95)	−0.15(− 0.43,0.14)	−0.16(− 0.45,0.14)	0.38 (0.03,0.72)*	0.39 (0.02,0.76)*
Vitality	29.89 (3.46)	29.73 (3.56)	29.99 (3.15)	−0.17(− 0.62,0.28)	0.01(− 0.45,0.47)	0.09(− 0.47,0.64)	−0.03(− 0.61,0.55)
Total score	122.89 (11.94)	125.30 (11.99)	126.10 (12.48)	2.41 (0.83,3.96)*	2.70 (0.70,4.70)*	3.11 (1.18,5.04) ***	3.14 (1.54,4.75)***

^a^Obstetrician-led Maternity Care

^b^Midwife-led Care at Delivery

^c^Continuity of Midwife-led Care

^d^cOR indicates crude odds ratio

^e^aOR indicates adjusted odds ratio (adjusted for hospitals, maternal age, education, vacation, method of payment, mode of birth, gestational age at delivery and birth weight)

*p < 0.05, **p < 0.01, ***p < 0.001

## Discussion

The principles of the midwife-led service were to provide a home-like environment, minimize intervention, facilitate normal birth, improve women’s birth experiences and enhance the role of midwives. The present study provided supportive clinical evidence on the effectiveness of the midwife-led maternity services for inexperienced primiparous women under China’s one-child policy. Other than increased vaginal birth and breastfeeding rates, the continuity of midwife-led service has contributed to improving the maternal wellbeing and satisfaction in low-risk primiparous women with limited experience under China’s one-child policy.

This study demonstrated that low-risk primiparous women who received the CMC or MCD services were less likely to undergo caesarean section than those who received the standard obstetrician-led birth care. These results supported previous studies that showed continuity of midwife-led care with a focus on normal birth in a friendly supportive birth environment makes a significant difference on the mode of delivery [4, 10, 19, 23] For low-risk pregnant women, collaboration and support other than standard obstetric management allowed a greater number of spontaneous vaginal deliveries [28]. It has been reported that midwives could provide plenty of information regarding childbirth to childbearing women. Increased self-confidence brought about by the presence of midwives made childbearing women more confident in facing labour [29]. However, in most areas in China, obstetrician-led birth care remains the most common form of pregnancy healthcare management. This physician-based obstetric care model is more likely to turn birth from natural process to a contrived medical process. A pregnant woman is often considered as a patient, and is therefore more likely to receive frequent obstetric intervention [30]. Under these circumstances, normal child birth usually ends up as caesarean delivery.

Breastfeeding education from midwives also plays an important role in protecting and supporting breastfeeding women [31, 32]. In our study, the continuity of midwife-led maternity service has been found to be linked to an increased rate of breastfeeding for inexperienced primiparous woman. However, the MCD service did not improve the breastfeeding rate. Breastfeeding knowledge from health professionals involves providing supportive attitudes and behaviors, but this knowledge must be accurate and thorough to effectively promote breastfeeding [33]. Multiple studies have shown that inconsistent and conflicting advices are detrimental to breastfeeding outcomes [34–36]. In the MCD model, the childbearing women may receive inconsistent information about breastfeeding during pregnancy because it is likely that they would have met a different obstetrician on each visit to the antenatal clinic at the out-patient department. Furthermore, they may also receive limited information regarding breastfeeding because the midwives in MCD care condition only provide services from labour to 2 h postpartum.

We observed that the continuity of midwife-led service was associated with increased women’s satisfaction. Similar to other studies on the continuity of midwifery care [37], women were more likely to feel satisfied with midwives in terms of information transfer, choices and decisions, and were more pleased with the antenatal and intra-partum care provided by the midwives compared to other models [38]. A recent review reported that women who received midwife-led continuity models of care were less likely to experience intervention and more likely to be satisfied with their maternity care compared to women who received other models of care [26]. The results of the current study were also consistent with a previous report that showed low-risk pregnant women in China were more likely to be satisfied with the care and support of midwives [39]. Many studies have showed that women in labour are more appreciative of reliable midwifery practice [40], especially health professionals who are open to listening, being honest, and can provide both physical and emotional support; in other words, the professionals who showed ability to care for the women’s needs during labour [41–44]. As previous studies have pointed out [45], the psychosocial aspects of birth care, as well as information and caregiver support are the most important factors associated with antenatal satisfaction; whereas the lack of support from midwives or other inappropriate antenatal services are often linked to dissatisfaction. However, our study did not observe any association between MCD and women’s satisfaction due to the limited service provided by the midwife from labour to 2 h postpartum.

In this study, we found that compared to the routine obstetrician-led birth care, the CMC service resulted in improved postpartum wellbeing, increased self-control and lower anxiety. However, MCD only had a positive influence on overall wellbeing. These results were similar to previous studies that showed midwife-led care could improve the maternal psychological status. Receiving support from midwives was one of the most effective methods to reduce the level of maternal anxiety [46]. Homer et al. also reported that women who had a familiar midwife during labour had a significantly higher sense of ‘control’ and a more positive childbirth experience compared to women with an unfamiliar midwife [10]. Cheung et al. [29] pointed out that there was a significant negative association between maternal anxiety and feelings of control during labour. Researchers [47] also reported that maternal feelings of control during labour might help in decreasing the level of anxiety during pregnancy.

### Strength and limitation

We conducted the present study on low-risk primiparous women in order to avoid other possible confounders such as severe complications during pregnancy, which may influence the wellbeing of multiparous women. In addition, the majority of childbearing women were primiparous under the rigorous birth control policy in urban areas of Mainland China. Therefore, it is difficult to compare the effectiveness of midwife-led maternity services between women under the one-child policy and women who are caring for their second child. However, since the one-child policy in China has been relaxed since 2016, we may be able to compare the effectiveness of midwife-led care between women under both policies in the future. However, caution should be made for the generalization of our results to the general obstetric population. Selection bias may exist in our study, including 29 women who refused to take part in our study at baseline, and 162 women with missing data or were excluded from the follow-up investigation. Moreover, women with a higher level of psychological well-being during the pregnancy are more likely to take part in our study and completing the follow-up investigation, which may affect the generalization of the study. A randomized intervention study may be necessary in the near future.

## Conclusions

Using a concurrent cohort study design, we found that the introduction of CMC significantly improved vaginal birth rate, breastfeeding rate, maternal satisfaction and overall wellbeing in pregnant women. However, MCD was only associated with vaginal mode of delivery and overall wellbeing. This study provides supportive evidence that CMC is the optimal healthcare service for the management of low-risk primiparous women under China’s one-child policy. Therefore a general implementation of CMC in Shanghai and across China should be encouraged. In our study, the majority of women (71.1%) still received the routine obstetrician-led birth care. Further efforts should be made to inform the public on the advantages of CMC in low-risk pregnant women.

## Abbreviations

aOR: Adjusted odds ratio
CI: Confidence interval
CMC: The continuity of midwife-led care
cOR: Crude odds ratio
CS: Caesarean sections
MCD: Midwife-led Care at Delivery
OMC: Obstetrician-led Maternity Care
VIF: Variance Inflation Factor
